# Supplementary material for: Rapid Response to Experimental Warming of a Microbial Community Inhabiting High Arctic Patterned Ground Soil
Source: Biology (Basel). 2022 Dec 14;11(12):1819. doi: 10.3390/biology11121819 (PMC9775327; doi:10.3390/biology11121819)
Supplement: Supplementary file 1 [file biology-11-01819-s001.zip › biology-2026928-supplementary.pdf]

Table S1. The 40 most frequent bacterial taxa recorded in soil[a]. Values in parentheses indicate the percentage of sequences in an OTU assigned to each taxonomic rank.

OTU	Phylum	Class	Order	Family	Genus
1	Proteobacteria (100)	α-Proteobacteria (100)	Sphingomonadales (100)	*Sphingomonadaceae* (100)	*Sphingomonas* (62)
2	Proteobacteria (100)	α-Proteobacteria (100)	Sphingomonadales (100)	*Sphingomonadaceae* (100)	m.d.
3	Proteobacteria (100)	β-Proteobacteria (100)	Burkholderiales (100)	*Sutterellaceae* (100)	m.d.
4	Proteobacteria (100)	α-Proteobacteria (100)	Caulobacterales (100)	*Caulobacteraceae* (100)	*Brevundimonas* (100)
5	Proteobacteria (100)	α-Proteobacteria (100)	Sphingomonadales (100)	*Sphingomonadaceae* (96)	m.d.
6	Proteobacteria (100)	α-Proteobacteria (100)	Sphingomonadales (100)	*Sphingomonadaceae* (100)	*Sphingomonas* (73)
7	Proteobacteria (100)	α-Proteobacteria (100)	Sphingomonadales (100)	*Sphingomonadaceae* (100)	*Sphingomonas* (96)
8	Gemmatimonadetes (100)	Gemmatimonadetes (100)	Gemmatimonadales (100)	m.d.	m.d.
9	Acidobacteria (100)	Blastocatellia (100)	Blastocatellales (100)	*Pyrinomonadaceae* (100)	m.d.
10	Verrucomicrobia (100)	Spartobacteria (100)	Chthoniobacterales (100)	*Chthoniobacteraceae* (100)	*Chthoniobacter* (74)
11	Proteobacteria (100)	α-Proteobacteria (100)	Sphingomonadales (100)	*Sphingomonadaceae* (100)	m.d.
12	Actinobacteria (100)	Acidimicrobiia (100)	Acidimicrobiales (100)	*Ilumatobacter* (100)	*Ilumatobacter* (100)
13	Proteobacteria (100)	α-Proteobacteria (100)	Rhizobiales (100)	*Parvibaculum* (100)	m.d.
14	Proteobacteria (100)	α-Proteobacteria (100)	Sphingomonadales (100)	*Sphingomonadaceae* (100)	m.d.
15	Proteobacteria (100)	α-Proteobacteria (100)	Sphingomonadales (100)	*Sphingomonadaceae* (100)	*Sandarakinorhabdus* (94)
16	Actinobacteria (100)	Actinobacteria (100)	Micrococcales (100)	*Intrasporangiaceae* (98)	*Oryzihumus* (95)
17	Proteobacteria (100)	γ-Proteobacteria (100)	Xanthomonadales (100)	*Xanthomonadaceae* (100)	m.d.
18	Bacteroidetes (100)	Sphingobacteriia (100)	Sphingobacteriales (100)	*Chitinophagaceae* (100)	*Ferruginibacter* (100)
19	Actinobacteria (100)	Rubrobacteria (100)	Gaiellales (100)	*Gaiellaceae* (100)	*Gaiella* (100)
20	Proteobacteria (100)	α-Proteobacteria (100)	Rhodobacterales (100)	*Rhodobacteraceae* (100)	*Amaricoccus* (97)
21	Actinobacteria (100)	Rubrobacteria (100)	Gaiellales (100)	*Gaiellaceae* (100)	*Gaiella* (100)
22	Actinobacteria (100)	Thermoleophilia (100)	Thermoleophilales (100)	*Thermoleophilaceae* (100)	m.d.
23	Proteobacteria (100)	α-Proteobacteria (100)	Rhodobacterales (100)	*Hyphomonadaceae* (98)	*Asprobacter* (98)
24	Actinobacteria (100)	Actinobacteria (100)	Pseudonocardiales (100)	*Pseudonocardiaceae* (100)	*Pseudonocardia* (100)
25	Proteobacteria (100)	α-Proteobacteria (100)	Sphingomonadales (100)	*Sphingomonadaceae* (96)	m.d.
26	Proteobacteria (100)	α-Proteobacteria (100)	Rhodospirillales (100)	*Acetobacteraceae* (100)	m.d.
27	Proteobacteria (100)	α-Proteobacteria (100)	Rhodospirillales (100)	*Acetobacteraceae* (100)	m.d.
28	Proteobacteria (100)	β-Proteobacteria (100)	Burkholderiales (100)	*Comamonadaceae* (100)	m.d.
29	Proteobacteria (100)	β-Proteobacteria (100)	Burkholderiales (100)	*Comamonadaceae* (100)	m.d.
30	Actinobacteria (100)	Rubrobacteria (100)	Gaiellales (100)	*Gaiellaceae* (100)	*Gaiella* (100)
31	Actinobacteria (100)	Nitriliruptoria (100)	Egibacterales (100)	*Egibacteraceae* (100)	*Egibacter* (100)
32	Actinobacteria (100)	Actinobacteria (100)	Pseudonocardiales (100)	*Pseudonocardiaceae* (100)	*Pseudonocardia* (100)
33	Gemmatimonadetes (100)	Gemmatimonadetes (100)	Gemmatimonadales (100)	*Gemmatimonadaceae* (100)	m.d.
34	Proteobacteria (100)	α-Proteobacteria (100)	Caulobacterales (100)	*Caulobacteraceae* (100)	*Phenylobacterium* (82)
35	Proteobacteria (100)	α-Proteobacteria (100)	Caulobacterales (100)	*Caulobacteraceae* (100)	*Aquidulcibacter* (100)
36	Acidobacteria (100)	Blastocatellia (100)	Blastocatellales (100)	*Blastocatellaceae* (100)	*Stenotrophobacter* (100)
37	Proteobacteria (100)	α-Proteobacteria (100)	Rhizobiales (100)	m.d.	m.d.
38	Acidobacteria (100)	Solibacteres (100)	Solibacterales (100)	*Bryobacteraceae* (93)	m.d.
39	Acidobacteria (100)	Solibacteres (100)	Solibacterales (100)	*Bryobacteraceae* (99)	m.d.
40	Bacteroidetes (100)	Sphingobacteriia (100)	Sphingobacteriales (100)	*Chitinophagaceae* (100)	*Ferruginibacter* (100)

[a]Note that none of the taxa could be classified at a percentage similarity of >97% to named species. *Abbreviation*: m.d., missing data.

Table S2. Soil bacterial alpha diversity metrics in control and treated soils.

	Control	OTC	Irrigation	OTC and irrigation
OTU richness	2366 ± 38	2299 ± 38	2292 ± 51	2333 ± 47
Shannon	6.34 ± 0.06	6.26 ± 0.05	6.28 ± 0.06	6.35 ± 0.05
Inverse Simpson	141 ± 14	124 ± 11	132 ± 10	155.7 ± 14
Chao1	4457 ± 63	4382 ± 111	4391 ± 117	4417 ± 167

Mean (n=10–11) values ± SEM are shown. Note that no main effects of OTCs or irrigation, or OTC × irrigation interaction effects, were recorded on the alpha diversity metrics.

Table S3. Outputs from PERMANOVA on Hellinger-transformed Bray-Curtis distances, testing for the effects of open top chambers (OTC), irrigation, block and the OTC × irrigation interaction on soil bacterial community composition. Analyses were based on 999 permutations.

	d.f.	*F* value	r^2 value	*P* value
OTC	1	1.59	0.038	0.152
Irrigation	1	0.79	0.020	0.558
Block	1	1.19	0.028	0.267
OTC × irrigation	1	0.78	0.018	0.551

Abbreviations: *d.f.*,degrees of freedom; OTC, open top chamber.

Table S4. Outputs from environmental fitting analyses on Hellinger-transformed Bray-Curtis distances, testing for the effects of volumetric soil water content (VSWC), open top chambers (OTC), irrigation, block and the OTC × irrigation interaction on soil bacterial community composition.

	NMDS1	NMDS2	r^2 value	*P* value
VSWC	-0.99537	-0.09609	0.0027	0.961
OTC	-0.68579	-0.72780	0.0044	0.901
Irrigation	-0.85519	-0.51832	0.0057	0.903
Control	0.99273	-0.12037	0.0450	0.407
OTC × irrigation	0.45716	-0.88938	0.0146	0.736
Block	0.47085	0.88222	0.0156	0.746

Abbreviations: NMDS, non-metric multidimensional scaling; OTC, open top chamber; VSWC, soil moisture concentration.

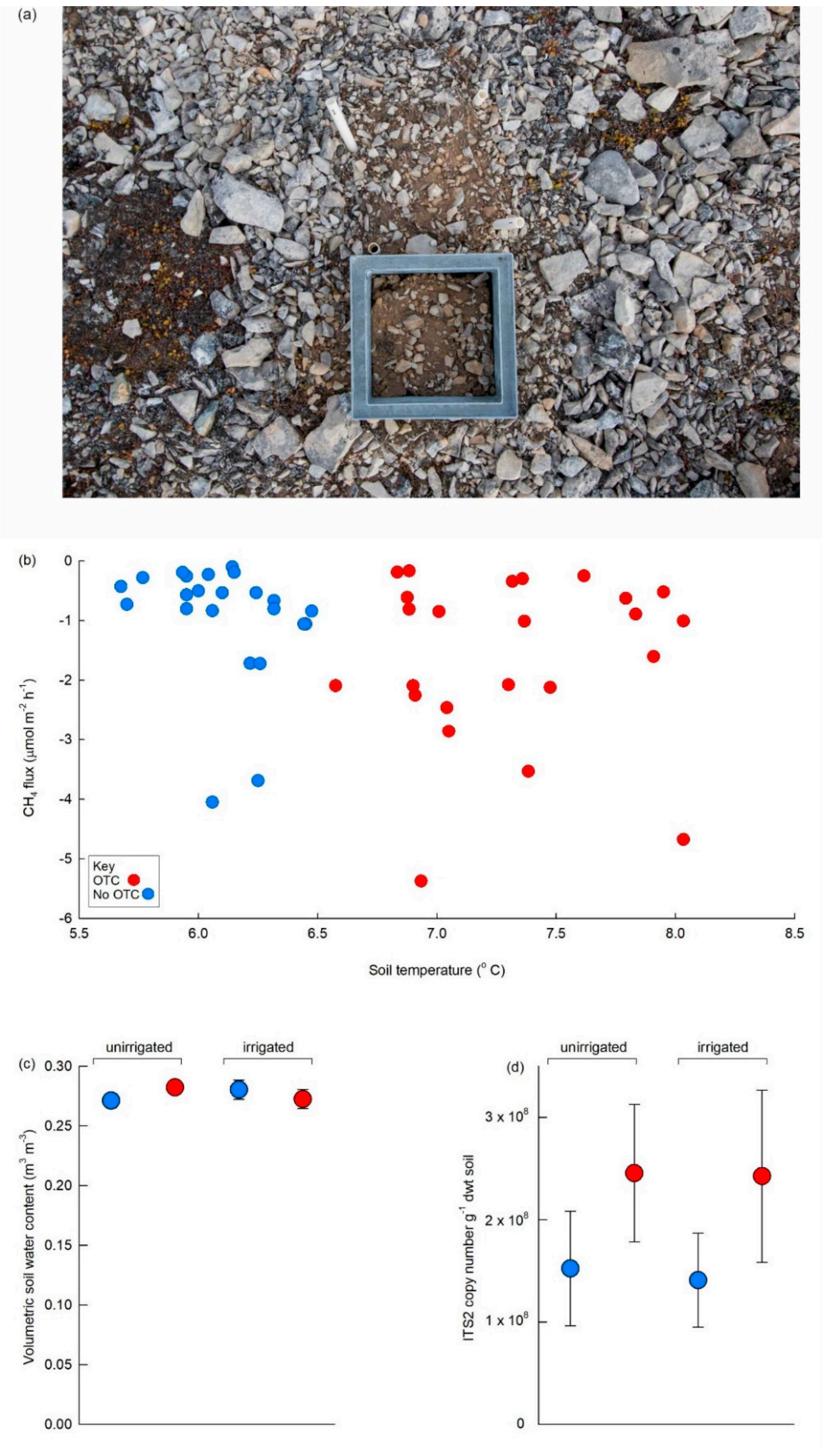

Figure S1. (a) One of the 48 plots in the experiment at Kongsfjordneset, which were centred on frost boils. Note the stainless steel frame (diameter 230 mm) with gutter, which was used to trap gases during flux measurements. (b) CH_4 flux as a function of soil temperature measured during sampling, (c) VSWC measured during sampling and (d) ITS2 copy numbers g^{-1} dry weight of soil. Values in (c) and (d) are means of 11–12 replicates, with bars showing SEM. Note that the bars for unirrigated soils in (c) are smaller than the means. Soil temperature and VSWC values are means of measurements over depths of 2 cm and 5 cm, and over the two days on which the measurements were made. See key in (b) for treatment notation.

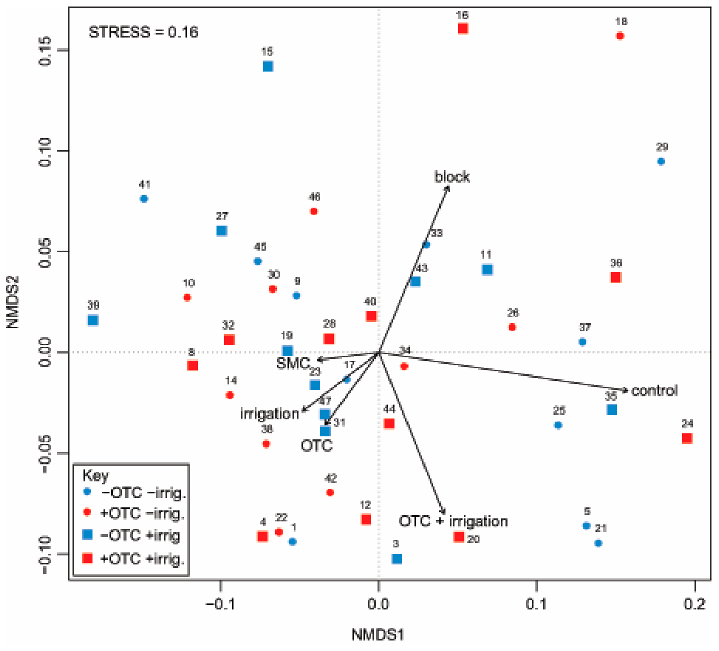

Figure S2. Ordination based on non-metric multidimensional scaling of Hellinger-transformed Bray-Curtis distances, illustrating the associations between the treatments, block and soil moisture concentration and bacterial community composition in each plot. See key for treatment notation. Arrows denote vectors for treatments, block and soil moisture concentration. Details of model outputs are shown in Tables S2 and S3. *Abbreviations*: OTC, open top chamber; irrig., irrigation; VSWC, volumetric soil water content.
